# Supplementary material for: A Likelihood Approach for Real-Time Calibration of Stochastic Compartmental Epidemic Models
Source: PLoS Comput Biol. 2017 Jan 17;13(1):e1005257. doi: 10.1371/journal.pcbi.1005257 (PMC5240920; doi:10.1371/journal.pcbi.1005257)
Supplement: S1 File — (TAR.GZ) [file pcbi.1005257.s014.tar.gz › HSPH_Online-SI-Revision/PF08/n10-mild/n10-mild_PF_table4.pdf]

1	R0	{1.25046, 6.80278}	{1.53617, 19.7618}	{{1.49778, 19.3526}, {1.56156, 20.}}	{22.8486, 190.495}	inf	141.	745.141	{696.161, 782.376}	428.469	0.722634	newly	135.	255.26	{231., 275.}	89.0815	0.275544	Reff	0.976984	0.880029	{0.852462, 0.904652}	9.92394	ar	3516.	7170.93	{6986., 7386.}	103.951
2	R0	{1.32013, 4.45847}	{1.64775, 16.221}	{{1.5009, 12.771}, {1.85918, 20.}}	{24.8172, 263.824}	inf	141.	911.855	{580.632, 1285.66}	546.706	0.79093	newly	183.	369.82	{299., 439.}	102.087	0.301392	Reff	0.815972	0.848455	{0.766051, 0.923638}	6.29188	ar	4420.	7709.34	{6981., 8560.}	74.4195
3	R0	{1.31785, 5.36796}	{1.726, 15.7604}	{{1.54917, 11.3264}, {1.9273, 20.}}	{30.9707, 193.602}	inf	276.	1194.16	{727.38, 1699.23}	332.665	0.619528	newly	309.	510.31	{441., 601.}	65.1489	0.214726	Reff	0.782671	0.933232	{0.864429, 1.01239}	19.2368	ar	4942.	8779.22	{8000., 9626.}	77.6451
4	R0	{1.38801, 6.16031}	{1.73156, 19.2858}	{{1.57885, 16.5703}, {1.88618, 20.}}	{24.7512, 213.065}	inf	192.	1145.05	{897.814, 1324.72}	496.38	0.768039	newly	196.	398.53	{338., 454.}	103.332	0.30538	Reff	0.811292	0.835451	{0.771863, 0.913243}	5.49184	ar	4790.	8312.35	{7608., 8831.}	73.5355
5	R0	{1.27955, 11.57}	{1.51951, 19.5253}	{{1.51185, 19.4183}, {1.53114, 19.5901}}	{18.7534, 68.7581}	inf	157.	134.449	{131.36, 137.295}	14.3637	0.0674205	newly	94.	45.59	{39., 53.}	51.5	0.317189	Reff	1.02351	0.626117	{0.621249, 0.630185}	38.8267	ar	3460.	7150.87	{7071., 7224.}	106.673
6	R0	{1.22184, 4.34395}	{1.59728, 16.4069}	{{1.50214, 13.3651}, {1.70628, 20.}}	{30.7276, 277.695}	inf	121.	940.613	{760.342, 1143.15}	677.366	0.88418	newly	169.	405.07	{359., 459.}	139.686	0.376771	Reff	0.87777	1.06405	{0.963775, 1.14366}	21.2221	ar	3524.	7730.76	{7232., 8278.}	119.375
7	R0	{1.23856, 4.59068}	{1.59151, 18.6962}	{{1.47805, 13.842}, {1.68243, 20.}}	{28.497, 307.264}	inf	125.	920.372	{704.45, 1087.14}	636.298	0.860312	newly	186.	338.56	{285., 385.}	82.0215	0.257279	Reff	0.844822	0.964775	{0.88764, 1.04215}	14.2405	ar	3855.	7796.51	{7168., 8221.}	102.244
8	R0	{1.34531, 10.6689}	{1.56011, 19.7292}	{{1.52529, 19.1921}, {1.59821, 20.}}	{15.9664, 84.9226}	inf	364.	783.88	{738.209, 826.96}	115.352	0.332697	newly	224.	268.77	{250., 285.}	19.9866	0.0783876	Reff	0.92221	0.860325	{0.836049, 0.890415}	6.71046	ar	4852.	7459.28	{7203., 7709.}	53.7362
9	R0	{1.31775, 5.19226}	{1.74931, 19.3559}	{{1.63049, 17.1715}, {1.86463, 20.}}	{32.7496, 272.784}	inf	255.	1242.45	{951.258, 1355.59}	387.234	0.683244	newly	288.	433.14	{367., 478.}	50.3958	0.175213	Reff	0.778658	0.885735	{0.819834, 0.939072}	13.7514	ar	4708.	8441.46	{7912., 8801.}	79.3003
10	R0	{1.25916, 6.89207}	{1.54401, 19.6355}	{{1.46095, 19.5543}, {1.57807, 20.}}	{22.6223, 184.899}	inf	145.	808.7	{696.115, 864.105}	457.724	0.742901	newly	135.	279.51	{238., 306.}	107.044	0.31412	Reff	0.888841	0.887606	{0.854956, 0.917511}	2.40987	ar	3823.	7469.21	{7059., 7729.}	95.3756
11	R0	{1.20377, 5.46566}	{1.52392, 18.3463}	{{1.44254, 13.2667}, {1.6121, 20.}}	{26.5959, 235.665}	inf	117.	763.929	{547.259, 919.742}	552.931	0.808735	newly	147.	291.39	{255., 326.}	98.2245	0.294953	Reff	0.911254	1.03829	{0.932421, 1.13364}	14.2688	ar	3114.	7173.53	{6614., 7685.}	130.364
12	R0	{1.27878, 2.55804}	{1.76214, 13.11}	{{1.70169, 12.2419}, {1.80144, 13.6643}}	{37.7983, 412.501}	inf	51.	1171.13	{1090.72, 1234.39}	2196.33	1.35971	newly	100.	603.91	{569., 639.}	503.91	0.780498	Reff	0.804992	0.899401	{0.875611, 0.918038}	11.7279	ar	3930.	9677.56	{9464., 9856.}	146.248
13	R0	{1.41227, 5.45696}	{1.62505, 19.8138}	{{1.5679, 19.6139}, {1.68959, 20.}}	{15.0668, 263.092}	inf	198.	912.096	{810.756, 1006.41}	360.655	0.661544	newly	224.	310.05	{270., 345.}	38.4152	0.139266	Reff	0.81262	0.806603	{0.747269, 0.854179}	3.44773	ar	4822.	7878.43	{7606., 8208.}	63.3851
14	R0	{1.32917, 7.27593}	{1.57716, 19.6028}	{{1.52221, 18.9787}, {1.63405, 20.}}	{18.6578, 169.419}	inf	261.	859.403	{773.029, 946.854}	229.273	0.516041	newly	225.	295.48	{262., 322.}	31.3244	0.116926	Reff	0.839903	0.853025	{0.813642, 0.885816}	3.20478	ar	4758.	7569.57	{7259., 7792.}	59.0914
15	R0	{1.31629, 9.97481}	{1.563, 19.697}	{{1.52614, 19.085}, {1.59353, 20.}}	{18.7426, 97.4676}	inf	321.	860.394	{794.92, 939.57}	168.036	0.427464	newly	226.	304.83	{276., 331.}	34.9248	0.129057	Reff	0.982216	0.980627	{0.951003, 1.00884}	1.76645	ar	4543.	7444.65	{7146., 7723.}	63.8708
16	R0	{1.36735, 5.74166}	{1.65257, 18.1993}	{{1.49821, 13.9001}, {1.82598, 20.}}	{20.8594, 216.969}	inf	216.	1039.49	{737.936, 1298.47}	381.247	0.671886	newly	224.	386.45	{320., 447.}	72.5223	0.233654	Reff	0.874557	0.913775	{0.850703, 0.9975}	5.93899	ar	4516.	7795.38	{7066., 8436.}	72.6169
17	R0	{1.27524, 13.2396}	{1.52968, 19.8752}	{{1.51365, 19.4437}, {1.55457, 20.}}	{19.9527, 50.1197}	inf	156.	769.965	{741.671, 800.782}	393.567	0.693124	newly	82.	265.42	{242., 285.}	223.683	0.509344	Reff	1.15027	0.922619	{0.905763, 0.932108}	19.7908	ar	3731.	7149.6	{6951., 7358.}	91.6269
18	R0	{1.21884, 7.71545}	{1.54145, 19.7679}	{{1.5021, 19.4399}, {1.57568, 20.}}	{26.4689, 156.213}	inf	198.	804.508	{757.117, 855.447}	306.317	0.608414	newly	194.	276.81	{249., 303.}	42.6856	0.153246	Reff	0.931559	0.912942	{0.888921, 0.937433}	2.26157	ar	3080.	7338.23	{7129., 7563.}	138.254
19	R0	{1.34879, 6.71464}	{1.61522, 17.0062}	{{1.50277, 14.3196}, {1.77458, 20.}}	{19.7536, 153.271}	inf	164.	852.461	{657.361, 1075.32}	419.793	0.707768	newly	162.	337.26	{289., 383.}	108.185	0.315449	Reff	0.798079	0.847778	{0.799099, 0.90586}	6.41328	ar	4585.	7969.	{7393., 8661.}	73.8059
20	R0	{1.37284, 5.75036}	{1.58308, 16.347}	{{1.48291, 12.6329}, {1.78224, 20.}}	{15.3141, 184.278}	inf	225.	850.041	{577.653, 1228.58}	277.796	0.562976	newly	244.	350.41	{286., 419.}	43.6107	0.153112	Reff	0.87848	0.901667	{0.83627, 0.94785}	4.6993	ar	4750.	7418.11	{6705., 8255.}	56.1707
21	R0	{1.29938, 5.36419}	{1.62805, 16.5}	{{1.50012, 13.3639}, {1.78563, 20.}}	{25.2945, 207.595}	inf	178.	896.562	{688.53, 1102.82}	403.687	0.692574	newly	209.	367.43	{321., 417.}	75.8038	0.242584	Reff	0.808604	0.864513	{0.803876, 0.935977}	7.62825	ar	4337.	7658.83	{7084., 8229.}	76.5928
22	R0	{1.31882, 7.47626}	{1.54059, 19.7419}	{{1.48379, 19.2702}, {1.57529, 20.}}	{16.8157, 164.061}	inf	191.	787.88	{721.072, 853.423}	312.503	0.614582	newly	169.	271.97	{248., 300.}	60.929	0.205446	Reff	0.929504	0.888803	{0.847886, 0.912583}	4.37885	ar	3977.	7202.63	{6916., 7498.}	81.1071
23	R0	{1.26215, 3.46375}	{1.63269, 16.8414}	{{1.44431, 13.6608}, {1.80633, 20.}}	{29.3579, 386.22}	inf	114.	947.304	{691.552, 1209.31}	730.968	0.904124	newly	207.	383.18	{319., 443.}	85.1111	0.26424	Reff	0.848291	0.947026	{0.865556, 1.02548}	11.8396	ar	3607.	7962.84	{7241., 8638.}	120.761
24	R0	{1.16712, 4.19464}	{1.54901, 19.4394}	{{1.47748, 18.5112}, {1.64546, 20.}}	{32.7206, 363.433}	inf	82.	842.748	{754.704, 923.397}	927.741	1.00979	newly	105.	297.	{264., 328.}	182.857	0.450029	Reff	0.873473	0.936496	{0.863468, 0.978743}	7.69935	ar	3000.	7693.54	{7332., 8051.}	156.451
25	R0	{1.22751, 3.34517}	{1.66259, 16.872}	{{1.54702, 13.8061}, {1.82199, 20.}}	{35.444, 404.37}	inf	114.	1023.37	{786.345, 1219.53}	797.697	0.946408	newly	241.	430.79	{387., 480.}	78.751	0.250161	Reff	0.932662	1.13821	{1.04442, 1.27044}	22.039	ar	3086.	8158.06	{7515., 8900.}	164.357
26	R0	{1.18747, 5.88083}	{1.53459, 19.8071}	{{1.49134, 19.2685}, {1.57879, 20.}}	{29.2315, 236.808}	inf	107.	792.043	{754.922, 845.047}	640.227	0.868438	newly	102.	269.16	{249., 292.}	163.882	0.420139	Reff	0.802017	0.846518	{0.816489, 0.872485}	5.94376	ar	3614.	7366.22	{7141., 7609.}	103.825
27	R0	{1.29023, 4.76202}	{1.64036, 19.3811}	{{1.49312, 18.2674}, {1.7401, 20.}}	{27.1371, 306.992}	inf	148.	1018.26	{795.546, 1178.43}	588.015	0.832459	newly	192.	353.99	{307., 396.}	84.3698	0.263534	Reff	0.787814	0.851214	{0.78737, 0.915651}	8.43198	ar	4245.	7953.08	{7211., 8433.}	87.3517
28	R0	{1.2258, 12.9056}	{1.5154, 19.9979}	{{1.50844, 20.}, {1.51766, 20.}}	{23.6252, 54.9552}	inf	171.	82.0292	{79.6503, 82.9395}	52.0297	0.319521	newly	91.	26.67	{20., 34.}	70.6923	0.541047	Reff	0.929156	0.607337	{0.606344, 0.610293}	34.6356	ar	3639.	7743.48	{7651., 7814.}	112.791
29	R0	{1.34702, 6.29049}	{1.57943, 18.2111}	{{1.46317, 13.679}, {1.67933, 20.}}	{17.2536, 189.502}	inf	220.	858.992	{606.164, 1030.58}	290.451	0.580896	newly	234.	315.83	{265., 359.}	35.0983	0.127595	Reff	0.838924	0.872531	{0.806973, 0.930472}	6.14493	ar	4560.	7369.62	{6626., 7838.}	61.6145
30	R0	{1.37931, 6.78741}	{1.58468, 18.2462}	{{1.4603, 13.4815}, {1.67508, 20.}}	{14.8891, 168.824}	inf	216.	831.128	{518.01, 1059.62}	284.782	0.573566	newly	206.	303.79	{243., 358.}	47.4709	0.164314	Reff	0.813793	0.844035	{0.772706, 0.90647}	6.05743	ar	4667.	7471.72	{6774., 8028.}	60.0969
31	R0	{1.16062, 4.47529}	{1.62653, 18.3813}	{{1.54585, 14.5733}, {1.74695, 20.}}	{40.1436, 310.728}	inf	144.	988.203	{808.959, 1199.84}	586.252	0.830834	newly	207.	382.77	{306., 460.}	84.913	0.262099	Reff	0.868956	1.08645	{1.03588, 1.16271}	25.0288	ar	3037.	8052.95	{7514., 8568.}	165.161
32	R0	{1.37647, 18.7122}	{1.52647, 19.9365}	{{1.49451, 19.6971}, {1.56151, 20.}}	{10.8976, 6.54259}	inf	353.	123.199	{112.196, 135.85}	65.0995	0.458561	newly	116.	39.85	{33., 47.}	65.6466	0.468404	Reff	0.957197	0.601926	{0.593817, 0.608397}	37.1158	ar	4824.	7551.32	{7393., 7684.}	56.5365
33	R0	{1.35084, 6.08841}	{1.73312, 19.365}	{{1.58386, 19.0255}, {1.85425, 20.}}	{28.2996, 218.063}	inf	251.	1164.22	{961.559, 1287.94}	363.832	0.661253	newly	266.	404.07	{332., 452.}	51.906	0.178939	Reff	0.778759	0.823079	{0.759523, 0.894295}	6.85176	ar	4759.	8360.54	{7787., 8761.}	75.6785
34	R0	{1.36912, 6.316}	{1.56983, 19.2759}	{{1.48755, 18.773}, {1.63467, 20.}}	{14.66, 205.192}	inf	243.	863.199	{754.114, 972.512}	255.226	0.546248	newly	247.	303.52	{267., 341.}	22.996	0.0878723	Reff	0.921965	0.914726	{0.870134, 0.960341}	3.26518	ar	4537.	7240.78	{6762., 7603.}	59.594
35	R0	{1.35831, 7.09038}	{1.64765, 17.7029}	{{1.50961, 13.5886}, {1.81687, 20.}}	{21.3015, 149.675}	inf	210.	865.453	{624.23, 1067.42}	312.12	0.604765	newly	184.	326.59	{283., 373.}	77.4946	0.246477	Reff	0.809553	0.844301	{0.773049, 0.921584}	6.52758	ar	4667.	8021.3	{7402., 8641.}	71.8727
36	R0	{1.28315, 5.89938}	{1.65335, 17.0168}	{{1.55342, 14.5228}, {1.78598, 20.}}	{28.8509, 188.451}	inf	195.	964.257	{784.789, 1167.41}	394.491	0.686398	newly	206.	383.88	{335., 426.}	86.3495	0.268293	Reff	0.765399	0.886225	{0.823546, 0.952977}	15.786	ar	4460.	8152.94	{7590., 8683.}	82.8013
37	R0	{1.19303, 2.75113}	{1.5236, 15.211}	{{1.35427, 8.81068}, {1.68337, 20.}}	{27.7083, 452.901}	inf	59.	769.407	{416.406, 1021.72}	1204.08	1.09586	newly	124.	347.24	{289., 399.}	180.032	0.443655	Reff	0.868407	1.01122	{0.917336, 1.10155}	16.4452	ar	3142.	7216.32	{5912., 8110.}	129.673
38	R0	{1.36033, 7.8088}	{1.61162, 18.4127}	{{1.49318, 14.1497}, {1.69478, 20.}}	{18.4729, 135.794}	inf	289.	943.19	{646.647, 1126.22}	226.363	0.505032	newly	222.	347.36	{294., 396.}	56.4685	0.191793	Reff	0.859593	0.919973	{0.854133, 1.00009}	7.72337	ar	4941.	7865.65	{7177., 8365.}	59.1915
39	R0	{1.27353, 3.70845}	{1.64202, 16.2549}	{{1.54547, 12.8166}, {1.80424, 19.9859}}	{28.9343, 338.321}	inf	54.	921.549	{689.958, 1100.16}	1606.57	1.22437	newly	83.	384.84	{344., 429.}	363.663	0.663546	Reff	0.815441	0.900183	{0.851099, 0.947732}	10.4295	ar	3725.	8037.78	{7562., 8601.}	115.779
40	R0	{1.19983, 12.1486}	{1.48715, 19.606}	{{1.47123, 19.5113}, {1.5072, 19.6609}}	{23.9464, 61.3852}	inf	143.	66.9379	{65.2015, 68.2748}	53.1903	0.329749	newly	81.	21.81	{16., 27.}	73.0741	0.5775	Reff	0.938027	0.606598	{0.59376, 0.611124}	35.3326	ar	3619.	7300.99	{7177., 7400.}	101.741
41	R0	{1.20935, 2.67169}	{1.744, 17.0354}	{{1.65016, 14.668}, {1.84007, 18.7111}}	{44.2096, 537.628}	inf	33.	1386.89	{1215.29, 1507.53}	4102.69	1.61917	newly	73.	569.25	{531., 614.}	679.795	0.891225	Reff	0.803008	1.0392	{0.95008, 1.09304}	29.4134	ar	3429.	9513.56	{9155., 9791.}	177.444
42	R0	{1.23313, 4.74781}	{1.53506, 19.716}	{{1.47611, 19.1228}, {1.59966, 20.}}	{24.485, 315.266}	inf	154.	805.174	{705.656, 871.203}	422.84	0.717102	newly	207.	278.49	{250., 304.}	34.5362	0.127502	Reff	0.853203	0.901035	{0.851719, 0.942015}	6.13351	ar	3835.	7380.34	{7140., 7656.}	92.4469
43	R0	{1.26105, 2.39713}	{1.6962, 17.0368}	{{1.61392, 14.7485}, {1.80031, 20.}}	{34.5068, 610.717}	inf	51.	1237.23	{1035.26, 1543.82}	2325.95	1.37956	newly	120.	499.05	{447., 549.}	315.875	0.617653	Reff	0.808711	0.999442	{0.901607, 1.08305}	23.5845	ar	3723.	9365.18	{8874., 9927.}	151.549
44	R0	{1.36948, 8.88006}	{1.56248, 19.4803}	{{1.50462, 18.6889}, {1.61548, 20.}}	{14.0927, 119.372}	inf	313.	861.702	{762.024, 960.217}	175.304	0.436986	newly	232.	300.09	{260., 343.}	29.7198	0.110935	Reff	0.929192	0.917642	{0.863864, 0.959618}	3.30947	ar	4437.	7217.37	{6852., 7541.}	62.6633
45	R0	{1.34882, 3.3791}	{2.0008, 15.684}	{{1.88568, 12.3983}, {2.34078, 20.}}	{48.337, 364.147}	inf	128.	1530.57	{1178.46, 2011.68}	1095.75	1.07078	newly	208.	662.91	{604., 714.}	218.707	0.502425	Reff	0.796074	0.92494	{0.881972, 0.988146}	16.1877	ar	4422.	9745.94	{9309., 10467.}	120.397
46	R0	{1.23774, 4.34917}	{1.61726, 16.8764}	{{1.48327, 13.5315}, {1.80257, 20.}}	{30.6626, 288.038}	inf	149.	955.347	{669.687, 1246.98}	541.172	0.796519	newly	217.	389.58	{317., 454.}	79.53	0.250137	Reff	0.844634	0.990692	{0.895682, 1.05824}	17.2925	ar	3685.	7666.32	{7048., 8415.}	108.041
47	R0	{1.34327, 4.78811}	{1.79785, 17.8162}	{{1.54284, 13.6682}, {2.06727, 20.}}	{33.8413, 272.092}	inf	220.	1273.33	{845.191, 1633.26}	478.785	0.748857	newly	304.	486.68	{411., 562.}	60.0921	0.201322	Reff	0.872454	0.955922	{0.881888, 1.0247}	9.895	ar	4517.	8298.4	{7213., 9084.}	83.7149
48	R0	{1.24366, 4.25814}	{1.58679, 19.0914}	{{1.48871, 18.0206}, {1.64326, 20.}}	{27.5904, 348.35}	inf	141.	893.451	{733.107, 1067.09}	533.653	0.795702	newly	200.	321.01	{265., 364.}	60.505	0.202354	Reff	0.862976	0.937566	{0.872899, 0.992595}	8.94263	ar	3678.	7323.81	{6848., 7712.}	99.1248
49	R0	{1.36017, 7.5415}	{1.5282, 19.6566}	{{1.48436, 19.0556}, {1.56257, 20.}}	{12.3538, 160.646}	inf	178.	793.545	{746.076, 834.065}	345.812	0.64866	newly	164.	274.98	{254., 296.}	67.6707	0.223664	Reff	1.01999	0.866449	{0.849082, 0.885487}	15.0533	ar	4178.	7448.83	{7249., 7640.}	78.287
50	R0	{1.25333, 6.63578}	{1.55401, 19.5397}	{{1.4855, 18.926}, {1.66856, 20.}}	{23.9901, 194.46}	inf	109.	800.545	{703.723, 847.793}	634.445	0.864976	newly	93.	279.26	{252., 303.}	200.28	0.476412	Reff	0.878334	0.854063	{0.786794, 0.892878}	3.63788	ar	3401.	7709.15	{7400., 8010.}	126.673
